# Association between oral health-related quality of life and nutritional status among older adults in district of Kuala Pilah, Malaysia

**DOI:** 10.1186/s12889-019-6867-1

**Published:** 2019-06-13

**Authors:** Tanti Irawati Rosli, Yoke Mun Chan, Rahimah Abdul Kadir, Tengku Aizan Abdul Hamid

**Affiliations:** 1 Department of Dental Public Health, Faculty of Dentistry, Universiti Kebangsaan Malaysia, Jalan Raja Muda Abdul Aziz, 50300 Kuala Lumpur, Wilayah Persekutuan Malaysia; 2 Malaysian Research Institute on Ageing, Universiti Putra Malaysia, UPM, 43400 Serdang, Selangor Malaysia; 3 Department of Nutrition and Dietetics, Faculty of Medicine and Health Sciences, Universiti Putra Malaysia, UPM, 43400 Serdang, Selangor Malaysia; 4 Faculty of Dentistry, Lincoln University College, No 2, Jalan Stadium, SS 7/15 Kelana Jaya, 47301 Petaling Jaya, Selangor Malaysia

**Keywords:** Quality of life, GOHAI, BMI, Nutritional status, Elderly

## Abstract

**Background:**

Poor oral health has an impact on food choices and intake of important nutrients among older population. The use of oral health-related quality of life instruments along with the clinical dental indicators can help to assess the oral problems that lead to nutritional problems in this group. This study aims to determine the association between oral health-related quality of life (OHRQoL) and nutritional status among a group of older adults in Kuala Pilah district, Malaysia.

**Methods:**

A cross-sectional study was carried out on 446 older adults aged 50 years and above from 20 randomly selected villages. Respondents were interviewed to collect information on their demographic characteristics and oral health perception, followed by physical examination to measure height, weight and body mass index (BMI) of respondents. The validated Malay version of General Oral Health Assessment Index (GOHAI) was used to measure OHRQoL.

**Results:**

About one-third (35.8%) of the respondents had normal BMI. Majority of the respondents were overweight (40.4%) and obese (19.9%), while only a small proportion was underweight (3.9%). Mean GOHAI score was 53.3 (SD = 4.7), indicating low perception of oral health. About 81.6% respondents had moderate to low perception of oral health. Logistic regression analysis showed a statistically significant association between the GOHAI and BMI scores (OR = 2.3; *p* < 0.01).

**Conclusions:**

Oral health-related quality of life was significantly associated with nutritional condition of respondents. Older adults with poor perception of their oral health were more likely to have unsatisfactory BMI compared to those who perceived their oral health to be good.

## Background

Like other developing countries, population ageing in Malaysia has become a major issue. It is documented that the proportion of population aged 60 years and above has risen from 6.3% in 2000 to 7.8% in 2010 and is expected to reach 10.7% in 2020 [1]. This phenomenon is a result of falling fertility and mortality rate, coupled with improvements in health system and therefore has brought about more people surviving into old age. Average life expectancy at birth of Malaysians has also increased for the past decades from 63.6 years in 1970 to 75.2 years in 2018 [2].

Increase in the proportion of the aged group means an increase in the prevalence of ill health and disabilities. Older people are associated with multiple acute and chronic diseases, greater medications usage as well as physiological changes of the ageing body systems [3, 4]. In addition to that, impaired nutritional state has also become a significant problem among elderly worldwide [5, 6]. In Malaysia, findings from the recent National Health and Morbidity Survey, NHMS, in 2015 showed that approximately 52.35% of older adults aged 60 years and above were overweight and obese [7].

Existing evidence indicated that several factors may have influence on nutritional status and healthy eating habit among elderly in the community. Oral health, as one of the associated factors, plays an important role in nutritional intake of older people. Poor oral health may expose the group at risk to underweight [8] or obese [9]. Reduced number of teeth and posterior occluding teeth among this group also had shown to affect their chewing ability leading to altered food choices and compromised nutritional status [10]. In addition to the clinical indicators, the impact of poor oral health on dimensions of quality of life also has been more pronounced among older people. Compromised dentition can lead to functional limitation (trouble biting and chewing food), psychological impacts (uncomfortable eating in front of others), pain and discomfort (discomfort when eating) and behavioural impacts (limit kinds or amount of foods) [11].

In recent decades, researchers have included the subjective assessment of psychological and social impacts as part of the analysis of oral health and nutritional status. Therefore, the oral health-related quality of life (OHRQoL) instruments can complement the objective clinical measurements and be used as a predictor of malnutrition in the aged population [12]. The Geriatric Oral Health Assessment Index, GOHAI, is one of the OHRQoL measurements that has been widely used in studies on self-perception of oral health among older population [13]. The internal consistency and validity of GOHAI have been shown to be satisfactory in several languages like Arabic, Japanese and Malay studies [14–16]. Several researchers have also used GOHAI when assessing the association between the impacts of oral conditions to nutritional status, as each of the GOHAI dimension focus on problems related to diet and nutrition like trouble biting or chewing food, discomfort when eating, uncomfortable eating in front of people and limit kinds or amounts of food [12, 17].

Body mass index, BMI, has been widely used for assessing the nutritional status of older adults in epidemiological studies [18, 19]. It is considered as the most practical tool because of its simplicity and low cost [20]. Furthermore, BMI classification of body size like overweight and obese had contributed to increase in awareness of fatness problem, leading to more successful epidemiological and intervention research related to health outcomes [21].

As both oral health and nutritional status are strongly related to healthy behaviours, it is hypothesised that those who have poorer oral health may be less likely to be conscious about their diet. Therefore, the aim of this study was to assess the association between oral health-related quality of life and nutritional status among a group of community-dwelling older adults in the district of Kuala Pilah.

## Methods

This cross-sectional study was conducted in the district of Kuala Pilah, Negeri Sembilan. The state of Negeri Sembilan is located at the west coast region of Peninsular Malaysia. Negeri Sembilan comprised of seven districts and is known to have a heterogeneous population in terms of ethnic groups and rural-urban distribution. Based from the 2010 Census of Malaysia, 19.1% of the population in Negeri Sembilan are older adults aged 50 years and above [1]. The district of Kuala Pilah was chosen as it has the highest percentage of older adults more than 50 years of age (25.8%; *n* = 16,499) among other districts in Negeri Sembilan.

Individuals aged 50 years and above, Malaysian, had been living in the selected areas for at least one year and could communicate clearly in Bahasa Malaysia were included in this study. Global studies on health and aging in several lower and upper-middle countries have also included aging adults aged 50 years and above as their respondents [22]. Those who were mentally ill and with other conditions that could affect the history taking and anthropometric measurements were excluded.

Based on a formula [23] used to calculate the adequate sample size in cross-sectional studies, n = z^2^ x P(1-P)/d^2^, where n is the sample size, z is the level of confidence at 95%, P is the estimated prevalence (51% prevalence of unsatisfactory body mass index among older adults) [24], and d is precision of 5%, the estimated sample size was 384. With estimation of 15% non-response subjects, a sample size of 442 was required for this study.

Respondents were recruited using a two-stage cluster sampling. Two sub-districts of Kuala Pilah, Pilah and Johol, were included for sample selection and from each sub-district, 10 villages were randomly selected. Both the sub-districts have similar socio-economic background as well as the number of older adults residing in the areas. Through the head villages, all the older adults within the selected villages were invited to participate in a health and dental screening programme at their respected community halls. Prior to the interview and examination, respondents were briefed on the aim and conduct of the study. Those who agreed to participate and matched the selection criteria were included.

Approval to conduct the study was obtained from the Medical Research Ethics Committee, Faculty of Medicine and Health Sciences, Universiti Putra Malaysia (UPM/FPSK/PADS/T7-MJKEtikaPer/F01(IG_Mei(10)03) and all the respondents provided written informed consent. Data gathered included socio-demographic characteristics, oral health status, oral health-related quality of life and body mass index.

### Socio-demographic and oral health-related quality of life

Data on socio-demographic and oral health-related quality of life were attained from the face-to-face questionnaire interview. Demographic variables involved items such as gender, age, ethnic group, educational level, living arrangement and medical conditions like hypertension, coronary heart disease, diabetes, asthma and arthritis. The 12-item Malay version of GOHAI was used to measure the oral health-related quality of life of the respondents in the past 3 months [16]. It consisted of three dimensions: physical function (4 items), psycho-social function (5 items) and, pain and discomfort (3 items). A five point Likert scale (always, often, sometimes, seldom and never) was used for measurement of the GOHAI index. The total GOHAI score was determined by summing the final score of each 12 items, ranged from 12 to 60. Higher score indicates better oral health-related quality of life. The scales for three of the positively worded items in the GOHAI (able to swallow comfortably, pleased with appearance of teeth and able to eat any kind of food) were reversed so that a higher score can reflect better self-reported oral health. For the analysis in this study, a dichotomous category of GOHAI was used whereby older adults with a score of 57 and more were identified as having ‘high perception’ of oral health, while those who scored between 12 and 56 were identified as having ‘low perception’ [12, 17]. Oral examination was carried out to determine the number of teeth in the oral cavity (teeth present and total tooth loss). Only one examiner was involved with clinical oral examination and demonstrated high intra-examiner consistency (Kappa value of 0.96).

### Body mass index

BMI was used to assess the nutritional status of the respondents. Respondents were measured for their standing height and weight, and readings were then used to calculate their BMI [weight (kg) / height (m^2^)]. The measurements were based on the Third National Health and Morbidity Survey format for anthropometrics measurement [25]. Weight was measured using Tanita 318 scale. The scale was checked for accuracy and calibrated prior to the start of the study. The respondents were asked to remove heavy items of clothing e.g. shoes and jackets, any heavy jewellery, keys and coins. After the scale was switched on and the zero reading displayed, the respondent was asked to stand on the scale. Weight was then recorded to the nearest 0.1 kg. The respondent was required to step off the scale and the first reading from the scale’s memory was cleared. The procedure was repeated and a second measurement made for an average reading. Height was measured using the SECA 206 portable body meter. The respondents were requested to stand straight, their feet together, arms loosely at their side, and with the head and back straight against the vertical surface of the wall. The respondent’s head was positioned in the Frankfort plane horizontally and once the correct position was achieved, the head plate was lowered until it touched the top of the respondent’s head. The measurement of height was recorded to the nearest 0.1 cm. The procedures were repeated and a second measurement made for an average reading. The WHO 2000 classification [26] was used to classify BMI into underweight (≤18.4 kg/m^2^), normal (18.5–24.9 kg/m^2^), overweight (25.0–29.9 kg/m^2^) and obese (≥30.0 kg/m^2^). BMI was further classified into two groups, satisfactory and unsatisfactory. Those who were underweight, overweight and obese were categorized as having unsatisfactory BMI.

Statistical analysis was performed using SPSS version 22.0. Descriptive statistics were determined, such as means and standard deviation (SD) for continuous variables and frequency and percentages for categorical variables. Associations between categorical variables were assessed with the Chi-square test of independence. The significance level was set at *p* < 0.05. A multiple logistic regression was constructed to evaluate the ability of GOHAI to predict unsatisfactory nutritional status among the older adults. BMI (satisfactory and unsatisfactory) was the dependent variable, while age group (50–69/70 & above), self-reported chronic disease (yes/no), dental status (dentate/edentate) and total GOHAI score (high/low) were the independent variables included in the model. Confidence interval (CI) at 95% of the odds ratio (OR) and *p*-value of the association were obtained to make inferences to the study population.

## Results

A total of 446 older adults participated in the study with 42.6% were men and 57.4% were women. The mean age was 63.2 years (SD 9.6). Majority (41.0%) of the respondents were in the age group of 50–59 years while only 4.9% in the oldest age group (> 80 years old). In relation to formal educational level, 9.6% had never attended school, 84.8% were with primary and secondary level of education and only a small proportion (5.6%) had tertiary education level. About 58.1% reported that they suffered from at least one medical condition.

The mean GOHAI score was 53.3 (SD 4.7). The mean score in those with satisfactory BMI was 53.9 (SD 4.9) and 52.9 (SD 4.5) in unsatisfactory BMI (*p* < 0.05). High proportion of the respondents (74.2%) had poor perception (GOHAI < 57) of oral health. Only 8.1% obtained a maximum score of 60, indicating no impact from oral conditions.

The mean BMI was 26.3 kg/m^2^ (SD 4.6). Majority of the respondents were overweight and obese, 40.4 and 19.9% respectively, 35.8% had normal body mass index and only 3.9% were underweight. Table 1 presents the demographic, oral health and nutritional characteristics of the older adults.

**Table 1:** Demographic characteristics, dental status and BMI of the study population

Items	*n* (%)
Sex	
Male	190 (42.6)
Female	256 (57.4)
Age group	63.2 (9.6)^a^
50–59	183 (41.0)
60–69	133 (29.8)
70–79	108 (24.2)
80 & above	22 (4.9)
Education level	
No formal education	43 (9.6)
Primary & secondary	378 (84.8)
Tertiary	25 (5.6)
Medical condition	
Yes	259 (58.1)
No	187 (41.9)
Dental status	
Dentate	356 (81.7)
Edentate	80 (18.3)
Total GOHAI score	
High perception	115 (25.8)
Low perception	331 (74.2)
Body mass index (BMI)	
Normal	157 (35.8)
Underweight	17 (3.9)
Overweight	177 (40.4)
Obese	87 (19.9)

^a^mean (SD)

Table 2 summarizes the differences between satisfactory and unsatisfactory BMI by socio-demographic characteristics and dental status. Younger age group and with medical problems were significantly associated with having unsatisfactory BMI (*p* < 0.05). A significant association was also observed between total GOHAI score and BMI (*p* < 0.001).

**Table 2:** Association between demographic characteristics and dental status with BMI

Variables	Satisfactory BMI *n* (%)	Unsatisfactory BMI *n* (%)	*p*-value
Sex			
Male	68 (43.3)	117 (41.6)	0.734
Female	89 (56.7)	164 (58.4)	
Age Group			
50–59	56 (35.7)	123 (43.8)	0.010*
60–69	43 (27.4)	89 (31.6)	
70–79	45 (28.6)	62 (22.1)	
80 & above	13 (8.3)	7 (2.5)	
Education level			
No formal education	16 (10.2)	27 (9.6)	0.407
Primary & secondary	129 (82.2)	241 (85.8)	
Tertiary	12 (7.6)	13 (4.6)	
Medical condition			
Yes	80 (51.0)	174 (61.9)	0.026*
No	77 (49.0)	107 (38.1)	
Dental status			
Dentate	122 (78.2)	228 (83.8)	0.147
Edentate	34 (21.8)	44 (16.2)	
GOHAI score			
High perception	56 (35.7)	55 (19.6)	< 0.001*
Low perception	101 (64.3)	226 (80.4)	

*Significant at *p*-level < 0.05

Table 3 shows the differences between respondents with satisfactory and unsatisfactory BMI for the percentage of responses for each of the GOHAI item. Compared to respondents who had satisfactory BMI, those with unsatisfactory BMI restricted more frequently the types of food they take and experienced more problems with biting or chewing (*p* < 0.01). Results in Table 3 also shows that respondents with unsatisfactory BMI were more frequently worried and self-conscious about their teeth, gums and dentures, used medications to relieve their pain and unable to eat any kinds of foods. Nevertheless, no significant differences were observed.

**Table 3:** Percentage of responses of to each item of GOHAI based on BMI

GOHAI items	Satisfactory BMI			Unsatisfactory BMI			p
	1	2	3	1	2	3	
Physical function							
Limit the kinds of food	14.0	40.8	45.2	14.2	56.2	29.5	0.003*
Trouble biting or chewing	15.9	41.4	42.7	15.7	56.5	27.8	0.003*
Able to swallow comfortably	94.9	5.1	0	95.7	4.3	0	0.812
Unable to speak clearly	0.6	10.8	88.6	1.1	8.9	90.0	0.733
Psychosocial							
Limit contacts with people	0.6	10.8	88.6	0.4	8.9	90.7	0.733
Pleased with appearance of teeth	100.0	0	0	99.6	0.4	0	0.642
Worried about teeth, gums or denture	0	15.3	84.7	2.1	12.8	85.1	0.149
Self-conscious of teeth, gums or denture	0	13.4	86.6	0.7	12.1	87.2	0.535
Uncomfortable eating in front of others	0.6	11.5	87.9	0.4	9.3	90.3	0.692
Pain/Discomfort							
Able to eat any kind of food	73.2	26.1	0.6	67.3	32.0	0.7	0.426
Use of medication to relieve pain	1.3	15.9	82.8	2.8	17.1	80.1	0.531
Sensitive to hot, cold or sweet foods	7.0	26.8	66.2	4.6	24.6	70.8	0.463

1-Always & often 2-Sometimes & seldom 3-Never

*significant at *p*-level < 0.05

A multiple logistic regression was constructed to evaluate the ability of GOHAI to predict unsatisfactory nutritional status among the older adults. The Hosmer and Lemeshow goodness-of-fit test was used to assess whether the final model accurately fits the data used. After adjusting for age, medical condition and dental status, a significant association was found between total GOHAI score and BMI. Respondents with lower total GOHAI score (low perception of oral health) were more likely to experience unsatisfactory BMI (Adjusted OR = 2.31; 95% CI 1.46, 3.64; *p* < 0.01) (Table 4).

**Table 4:** Multiple logistic regression analysis for the association between total GOHAI score and unsatisfactory BMI

Variables	Adjusted Odd Ratio^a^	95% of CI for OR	*p* value
Age group^b^			0.011*
50–69	1.84	1.15, 2.94	
70 & above	1.0	–	
Medical condition^b^			0.016*
Yes	1.67	1.10, 2.52	
No	1.0	–	
Dentate status^b^			0.43
Dentate	1.0	–	
Edentate	0.80	0.47, 1.39	
Perception of oral health (GOHAI score)			
High	1.0	–	< 0.001*
Low	2.31	1.46, 3.64	

^a^The multiple logistic regression model is reasonably fit with Hosmer-Lemeshow goodness of fit. (χ^2^(8) = 10.968; *p* = 0.204), area under ROC = 0.67

^b^There is no significant interaction between age group, medical condition and dentate status (Controlled variables)

## Discussion

In this study, older adults with poor perception of their oral health had unsatisfactory BMI. Consistent results were also observed in other studies that evaluated the association between OHRQoL and nutritional status in older population [8, 17]. Respondents with poor GOHAI score were shown to be at risk of nutritional deficiencies. However, most of these studies used Mini-Nutritional Assessment (MNA) for measuring nutritional status, thus comparison can only be made on the risk of nutritional deficit and not the actual BMI score.

Association between impacts of oral health on the quality of life with nutritional status have been established in several studies [8, 27]. As most of the impacts were related to eating and chewing, impaired nutrients intake could be evident among the elderly group. GOHAI evaluates the impact of oral conditions on the dimensions of oral health-related quality of life. The index focuses mainly on mastication-related problems in all dimensions – limit on types and amount of food, difficulties with biting or chewing food, discomfort when eating or swallowing and feeling uncomfortable eating in front of others. A high prevalence of these problems was reported among all the GOHAI items [14, 16, 28] and therefore would present a strong association between the OHRQoL measure and nutritional status. Similarly, in the present study, older adults with unsatisfactory BMI had shown significant difference in relation to the frequency of restriction of food and chewing problems. In Malaysia, rice, fish, green vegetables, bread and local *kuih* were among the common food consumed in a day [29]. As most of older adults have compromised dentition, avoidance of these food due to limited number of teeth is more likely to occur.

Cousson et al in 2012 examined two groups of elderly, fully dentate and complete denture wearers, to identify the factors associated with risk of malnutrition. Findings revealed that fully dentate elderly individuals have better GOHAI score, indicating less impact of oral conditions on their oral health-related quality of life. Elderly with least risk of malnutrition were those fully dentate, living in a couple and have high mean GOHAI score [30]. Similar findings were highlighted in an Australian study in which older adults with better perception of oral health (higher GOHAI score) were among those at least risk of malnutrition (lower MNA score) [31]. As both oral health and nutritional status are strongly related to healthy behaviours, these findings may also suggest that those who have poorer oral health may be less likely to be conscious about their diet. However, a causal relationship between malnutrition and OHRQoL could not be explained as both studies were cross sectional in design, thus future longitudinal research are required to address the association.

Clinical measurements like reduced number of teeth and occluding teeth, dental caries and periodontal disease have been commonly linked to nutritional deficit [32]. Decline in the oral health characteristics could lead to negative self-perception of oral health as defined by the GOHAI [33]. Beside the above mentioned clinical parameters, it can also be suggested that poor self-perception on oral health was one of the variables associated with nutritional deficiencies. Therefore, it is evident that OHRQoL instruments can complement the objective clinical measurements and be used as a predictor of malnutrition especially in the aged population.

Contrarily, no association was found between OHRQoL measurements and nutritional status among older adults in some studies [34, 35]. This may be explained by the various factors affecting food choices and intakes among elderly, like general health, socioeconomic factors, taste and control over food preparation [36]. There may also be lack of knowledge on nutritive value of foods consumed among the older individuals, putting them still at risk of malnutrition regardless of their oral perceptions. Furthermore, the sample size in the previous study was small and therefore results might not be parallel to the present study.

Findings from this study also highlighted that majority of the respondents were overweight and obese (40.4 and 19.9% respectively). One of the possible reasons may be due to the level of education among respondents in which 55.1% of them have only primary or without formal education. Level of education has influence on elder’s nutrition knowledge and practice, giving impact to their nutritional health [37]. It was reported that 73% of Malaysians elderly have poor nutrition knowledge and this was significantly correlated with educational status [38]. Similarly, poor oral health status was associated with low education level and may have positive impact on general and oral health-related quality of life [39].

In Malaysia, the National Oral Health Survey of Adults, NOHSA (2010) has reported the impacts of oral conditions on the oral health-related quality of life of the population studied. More than one quarter of the respondents (29.3%) had experienced at least one or more impact and the proportion increased with age [40]. Similar to the earlier NOHSA data in 2000, 54.3% of elderly aged 75 years and above complained of oral functional limitations. Among those with oral functional limitations, majority (19.6%) reported problems with chewing hard foods. Of those who reported problems with chewing, edentulous elderly without dentures had the most impact (85.7%) compared to edentate with dentures and dentate groups [41]. These unfavourable results for chewing-related problems should alert the dental practitioners of the possibility of nutritional problems especially in the aged populations. Despite of the high impact of oral conditions on OHRQoL, utilization of dental care service by the elderly remains low [41]. Similar report showed that utilization of oral care services among older people in Malaysia was the lowest compared to younger population. Further studies focusing on ways to eliminate barriers and encourage uptake of dental services need to be developed.

The study also indicated that older adults with unsatisfactory BMI significantly decreased with advancing age. This pattern is consistent with the local data of NHMS 2015 study in which the reason could be due to the fact that older individuals with unsatisfactory BMI, for example obesity, had died earlier because of chronic diseases related to the condition like cardiovascular diseases, thus leaving the non- obese individuals with a higher survival rate in the older age group [42].

Despite the associations observed, one of the limitation of this study was it could not establish a causal relation between OHRQoL and nutritional status as its cross-sectional design. Longitudinal studies may be needed to further explore the relationship. As cluster sampling technique was applied in the study, sampling error could also occur and lead to over- or under-represented in the final conclusion. Nevertheless, both selected sub-districts have similar socio-economic background as well as the number of respondents. Another limitation was the use of self-reported tools to measure the oral health-related quality of life among the respondents. The GOHAI has potential bias as they rely on respondents’ memory and ability to identify the oral health impacts [43]. Furthermore, older people are associated with high prevalence of hearing and memory problems which may lead to additional sources of bias. Nevertheless, highly trained interviewers were used to conduct such procedures to improve reliability of the study. Moreover, it can yield relevant information on the self-perceived oral impacts among older population especially in relation to masticatory ability. To date, local data on self-perception of oral health among the older age group is very scant, so therefore it could add to the limited literature on oral health and nutrition at the community level.

## Conclusion

In conclusion, this study suggested that older adults with poor perception of oral health were more likely to have unsatisfactory BMI. The application of oral health-related quality of life instruments together with objective clinical measurements need to be emphasized as it could be utilised as oral health predictors that might lead to impaired nutrition in the older population.

## Abbreviations

BMI: Body mass index
GOHAI: Geriatric Oral Health Assessment Index
NHMS: National Health and Morbidity Survey
NOHSA: National Oral Health Survey of Adults
OHRQoL: Oral health-related quality of life
